# Identification and Mechanism Research of Oxidative Stress-Related Biomarkers in Oral Lichen Planus

**DOI:** 10.3390/biomedicines14020420

**Published:** 2026-02-13

**Authors:** Qiao Peng, Xiangwen Bu, Shixian Zang, Ning Duan, Xiang Wang, Wenmei Wang

**Affiliations:** Nanjing Stomatological Hospital, Affiliated Hospital of Medical School, Institute of Stomatology, Nanjing University, 30 Zhongyang Road, Nanjing 210008, China; pengqiao0605@163.com (Q.P.); xwb230901@outlook.com (X.B.); zangshixian715@163.com (S.Z.); smallbird@sina.com (N.D.)

**Keywords:** oral lichen planus, oxidative stress, biomarkers, drug prediction, single-cell RNA sequencing

## Abstract

**Background:** Oxidative stress (OS) plays an important role in oral lichen planus (OLP) development; however, the precise functions of the genes associated with OS (OSRGs) remain unclear. This study aimed to identify and characterize OS-linked molecular markers in OLP. **Methods:** Data were obtained from the GSE38616 and GSE211630 datasets, along with 467 OSRGs. Candidate genes were identified by cross-referencing differentially expressed genes with OSRGs. Biomarkers were then selected through a protein–protein interaction network analysis using Cytoscape. Functional enrichment analysis, regulatory network mapping, therapeutic compound prediction, molecular docking simulations, and RNA modification profiling were also performed. Single-cell RNA sequencing was used to characterize biomarker distribution among the distinct cell populations. Gene expression was validated using quantitative real-time PCR (qRT-PCR). **Results:** Five genes emerged as key biomarkers: TGFB1, KLF4, TNF, NQO1, and MMP9. Functional enrichment analysis revealed that these markers are involved in immune regulatory pathways between lymphoid and nonlymphoid cellular compartments. Network analysis identified hsa-miR-449a and hsa-miR-449b-5p as potential regulators of NQO1 and KLF4. Pharmaceutical screening identified several potential therapeutic compounds, such as meropenem anhydrous and hydroxyurea, which exhibit targeted binding affinity for key biomarkers. Docking simulations indicated robust binding interactions (binding energies < −5 kcal/mol) for most compound–biomarker combinations, excluding the KLF4–hydroxyurea pairing. In addition, putative m6A methylation sites were identified in the TNF, KLF4, and TGFB1 transcripts. Single-cell analysis identified T lymphocytes as the primary cell type of interest, with TGFB1 expression increasing progressively during T-cell maturation. Validation by qRT-PCR confirmed the transcriptomic results, demonstrating elevated expression of TGFB1, TNF, and MMP9, along with reduced NQO1 expression in OLP tissues. **Conclusions:** TGFB1, KLF4, TNF, NQO1, and MMP9 were identified as potential OS-associated biomarkers in OLP. These findings provide insights into disease mechanisms and reveal potential therapeutic targets.

## 1. Introduction

Oral lichen planus (OLP) is a chronic inflammatory condition of the oral mucosa mediated by T cells. Its prevalence ranges from 0.1% to 4% in the general population [1,2]. This disease predominantly affects middle-aged women and manifests through diverse presentations, including reticular, erythematous, and erosive patterns [3]. As a potential oral malignant disorder, OLP has a malignant transformation rate of approximately 1% over 7 years [4,5]. Its epidemiology, pathophysiology, diagnosis, therapy, management, and immune mechanisms have been extensively studied [2]. Currently, the primary treatment goal is symptom control using corticosteroids, calcineurin inhibitors, and photodynamic therapy [6,7]; however, there is no definitive cure for OLP. The disease may be associated with delayed healing, frequent recurrence, severe pain, and resistance to therapy, particularly in erosive lesions [8]. Therefore, identifying novel biomarkers for the precise prevention and control of OLP is essential.

Oxidative stress (OS) is a condition in which excessive reactive oxygen species generated by multiple factors disrupt cellular homeostasis [9]. This imbalance results in oxidative tissue damage through various mechanisms, including DNA modification, lipid peroxidation, and protein oxidation [10]. OS has been documented in numerous systemic conditions, including cardiovascular disorders, neurodegenerative diseases, autoimmune disorders, and malignancies [11,12,13,14]. Increasing evidence indicates that OS plays a crucial role in OLP, as reflected by elevated levels of lipid peroxidation products, malondialdehyde (MDA), 8-hydroxy-2′-deoxyguanosine (8-OHdG), and advanced oxidation protein products (AOPPs) in saliva or serum [15]. Notably, increased expression of 8-OHdG is a key biomarker of oral carcinogenesis [16]. These findings suggest that OS plays a significant role in both the development and malignant transformation of OLP.

A comprehensive understanding of the OS landscape in OLP requires the functional categorization of relevant biomarkers. Although antioxidant enzymes are essential for maintaining redox balance, their expression patterns and roles in OLP progression remain unclear [17]. Similarly, immune regulators such as TGFB1 and TNF modulate reactive oxygen species; however, their synergistic interactions with OS in OLP have not been systematically analyzed [18,19]. Although MMP9-mediated tissue remodeling is associated with OS, its direct regulatory role in OLP pathogenesis remains unknown [20]. Furthermore, despite evidence that transcription factors such as KLF4 regulate OS-responsive genes, their specific mechanisms of interaction in OLP have not yet been elucidated [21].

In this study, core OS-related biomarkers in OLP were identified by integrating the GSE38616 dataset with 467 OS-related genes (OSRGs) through differential expression and protein–protein interaction (PPI) network analyses. Their putative mechanisms were subsequently explored using functional enrichment analysis and lncRNA–miRNA–mRNA network construction. Targeted therapeutic compounds and epigenetic features were examined through molecular docking and N6-methyladenosine (m6A) modification analyses. In addition, single-cell RNA sequencing data from the GSE211630 dataset were used to elucidate cell type–specific expression patterns and differentiation trajectories. Finally, the expression of these biomarkers was validated in clinical OLP samples using quantitative real-time PCR (qRT-PCR) to confirm their relevance to OLP pathogenesis.

To date, core OS-related biomarkers in OLP, and their regulatory mechanisms, cell type specificity, and therapeutic potential, have not been comprehensively identified. Therefore, an integrated strategy combining bulk transcriptomic profiling, single-cell RNA sequencing data, multidimensional bioinformatic analyses, and clinical sample validation was employed. This approach provided a cohesive framework encompassing systematic screening, mechanistic exploration, and experimental verification. The findings offer novel insights into OS-mediated OLP pathogenesis and establish a foundation for the identification of precise biomarkers and targeted therapeutic interventions.

## 2. Materials and Methods

### 2.1. Data Sources

OLP-specific datasets were obtained from the Gene Expression Omnibus (GEO) database (https://www.ncbi.nlm.nih.gov/geo/). The GSE38616 dataset (platform GPL6244) includes oral mucosal tissue samples from seven patients with OLP and seven healthy controls. In addition, the single-cell RNA sequencing (scRNA-seq) dataset comprises samples from five patients with OLP and one healthy control. Although these datasets provide valuable transcriptomic insights, the relatively small sample size—particularly the inclusion of only one healthy control in the scRNA-seq dataset—may limit statistical power and the ability to fully capture patient heterogeneity. A total of 467 OS-related genes (OSRGs) were identified from the literature [22] (Table S1).

### 2.2. Recognition and Functional Annotation of Candidate Genes

A PPI network was constructed using the STRING database (https://www.string-db.org) with a confidence score ≥ 0.4 and visualized using Cytoscape (v3.8.2). Core biomarkers were identified using the MCODE plugin in Cytoscape (v3.8.2) with the following parameters: degree cutoff = 2, node score cutoff = 0.2, K-core = 2, and maximum depth = 100. Spearman correlation analysis was performed using the *psych* package (v2.1.6) to assess relationships among biomarkers (|correlation coefficient| > 0.3, *p* < 0.05). Biomarker expression was validated in the GSE211630 dataset using the Wilcoxon rank-sum test (*p* < 0.05).

### 2.3. Identification of Biomarkers

A PPI network was constructed using the STRING database (https://www.string-db.org) with a minimum confidence score threshold of 0.4, followed by network visualization using Cytoscape software (v 3.8.2). Core biomarkers were identified by extracting densely connected modules using the MCODE plugin integrated into Cytoscape (v3.8.2). The algorithmic parameters were configured as follows: degree = 2, node score = 0.2, K-Core = 2, and maximum depth = 100. To assess relationships among the biomarkers, Spearman’s correlation analysis was implemented using the psych package (v 2.1.6), applying stringent criteria of |correlation coefficient| exceeding 0.3 combined with *p* < 0.05. Moreover, validation of the expression of these biomarkers was achieved through Wilcoxon rank-sum statistical testing using the GSE211630 dataset (*p* < 0.05).

### 2.4. Enrichment Analysis of Biomarkers

To systematically identify the biological functions of candidate biomarkers in OLP pathogenesis, GSEA was performed using the expression profiles within the GSE38616 dataset. The reference gene collection c2.cp.reactome.v7.0.symbols.gmt obtained from the MSigDB (https://www.gsea-msigdb.org/gsea/msigdb/) was used as the background reference set. Spearman’s correlation coefficients quantifying the relationships between biomarkers and all other detected genes were calculated using psych (v2.1.6). Next, individual GSEA was conducted for each biomarker using clusterProfiler (v 4.2.2), with significance criteria defined as |NES| > 1, FDR < 0.25, and Padj < 0.05. The top 5 most significantly enriched pathways (ranked by Padj) were visualized using enrichplot (v 1.18.3). OLP patient samples within GSE38616 were then dichotomized into high- and low-expression subgroups based on the median expression level of each biomarker. Using c5.all.v7.5.1.entrez.gmt from MSigDB as a reference set, GSVA was conducted using the GSVA package (v 1.42.0). Differentially activated biological pathways between the expression groups were identified using the limma package (v 3.54.0) with a criterion of |logFC| > 0.5, |t| > 2, and Padj < 0.05. Moreover, the GeneMANIA database (http://genemania.org) was used to predict functionally associated genes and construct a GGI network. The interaction probabilities between the candidate biomarkers and proteins encoded by co-expressed genes were computationally predicted through RF and SVM classifiers in the RPISeq database (http://pridb.gdcb.iastate.edu/RPISeq/). High-confidence interactions were defined by the dual criteria of RF > 0.8 and SVM > 0.8.

### 2.5. Construction of Regulatory Network and Disease-Association Analysis

Three databases, TargetScan Human 7.2 (http://www.targetscan.org/vert_72/), miRDB (http://mirdb.org/), and miRWalk 3.0 (http://mirwalk.umm.uni-heidelberg.de/, were used to predict miRNAs that target these biomarkers. Key miRNAs were identified by intersecting the predictions from all three databases. Using the miRNet database (https://www.mirnet.ca), upstream lncRNAs regulating these key miRNAs were identified, which enabled the construction of an lncRNA–miRNA–mRNA regulatory network. Given the potential for OLP to progress to oral squamous cell carcinoma (OSCC), the Comparative Toxicogenomics Database (CTD) (https://ctdbase.org/) was used to identify cancer-related diseases associated with these biomarkers and establish a disease–biomarker association network. To evaluate biomarker associations with OSCC, their expression in OSCC samples was analyzed using the Cancer Genome Atlas (TCGA) database (https://www.cancer.gov/). Heatmap visualization was done using the pheatmap package (v 1.0.12).

### 2.6. Drug Prediction and Molecular Docking

DGIdb (http://www.dgidb.org/) was used to predict drugs that target the candidate biomarkers. Drug–biomarker networks were visualized using Cytoscape (v3.8.2). Compound structures were retrieved from PubChem (https://pubchem.ncbi.nlm.nih.gov). Key compounds were selected by molecular weight and accessibility. The 2D structures (.SDF) for the compounds in PubChem and the protein structures (.PDB) from AlphaFold (https://alphafold.ebi.ac.uk/) were subjected to molecular docking using CB-Dock (v2.0). A binding energy < −5 kcal/mol indicated a favorable interaction.

### 2.7. RNA Modification and RNA-Binding Protein Analysis

N6-methyladenosine (m6A) modifications regulate gene expression. The SRAMP database (http://www.cuilab.cn/sramp/) was used to predict m6A modification sites within the biomarkers. The highest-scoring m6A modification site for each biomarker was subsequently visualized within the RNA secondary structure. Next, the ENCORI database (https://rnasysu.com/encori/index.php) was used to predict RBPs targeting the biomarkers, whereas the RPISeq database was used to calculate binding probabilities between the RBPs and biomarkers. Finally, the RBPsuite database (http://www.csbio.sjtu.edu.cn/bioinf/RBPsuite/) was used to identify RNA regions where the RBPs interact with the biomarkers.

### 2.8. scRNA-Seq Analysis

GSE211630 was processed using Seurat (v4.3.0). The cells and genes were filtered (min.cells = 3) with the following QC criteria: 200 < nFeature_RNA < 4000, 500 < nCount_RNA < 10,000, percent.mt < 25%. Data normalization was performed using the NormalizeData function. FindVariableFeatures identified the top 2000 HVGs for principal component analysis (PCA) using runPCA. Optimal principal components were selected using JackStraw and visualized with Elbowplot. UMAP clustering (resolution = 0.3) using FindNeighbors and FindClusters segmented the cells into distinct populations. FindAllMarkers identified characteristic markers, with clusters annotated using the CellMarker database (http://xteam.xbio.top/CellMarker/) (Table S2). DotPlot was used to visualize marker gene expression across all the cell types. ReactomeGSA (v1.16.1) was used for functional enrichment of the annotated populations.

### 2.9. Identification of Key Cells and Pseudotime Analysis

PCA dimensionality reduction selected the top 30 PCs from the HVGs for analysis. UMAP clustering using FindNeighbors and FindClusters (resolution = 0.3) generated distinct cell populations. FindAllMarkers (logFC > 0.25, min.pct > 0.25) identified cell-type markers that were annotated using the CellMarker database (e.g., T cells, B cells, and fibroblasts).

Five core biomarkers (TGFB1, KLF4, TNF, NQO1, and MMP9) were analyzed among the cell types through violin and dot plots. The Wilcoxon rank-sum test was applied to compare biomarker expression between OLP and controls within each lineage to identify key significantly enriched populations (*p* < 0.05).

Monocle3 (v1.3.7) was used to investigate the biomarker dynamics in key cells (T cells) through pseudotime trajectory analysis. The cells were ordered along developmental paths, with biomarker trends (e.g., TGFB1) plotted to elucidate their roles in T-cell differentiation and lineage specification in OLP.

### 2.10. qRT-PCR

A total of 37 oral mucosal tissue specimens were obtained from 23 OLP patients and 14 control subjects at Nanjing Stomatological Hospital, Affiliated Hospital of Medical School, Nanjing University. The average age of the OLP patients was 54.17 ± 11.35 years, whereas the control subjects had a mean age of 48.93 ± 9.11 years. The clinical characteristics for the study participants are listed in Table 1. Written informed consent was obtained from all participants. Ethical approval for this study was granted by the ethics committee of Nanjing Stomatological Hospital, Affiliated Hospital of Medical School, Nanjing University (approval number: NJSH-2023NL-096, registration date: 17 December 2023).

Total RNA extraction from 10 samples was done using TRIzol reagent (Ambion, Austin, TX, USA) based on standard protocols. The RNA was quantified using the NanoPhotometer N50. First-strand cDNA was synthesized using the SureScript kit on the S1000TM Thermal Cycler (Bio-Rad, Hercules, CA, USA). The primers are listed in Table S3. qRT-PCR was run on the CFX Connect system (Bio-Rad, USA) with the following parameters: 95 °C for 1 min (initial denaturation), 40 cycles of 95 °C for 20 s, 55 °C for 20 s, and 72 °C for 30 s. Expression levels were normalized to ACTIN using the 2^−ΔΔCT^ method. Excel and GraphPad Prism v5 were used for analysis and visualization (*p* < 0.05).

### 2.11. Statistical Analysis

Statistical analysis was performed using R (v 4.2.2). Given the relatively small sample sizes (*n* < 10 per group) non-parametric methods were employed to ensure statistical robustness. Specifically, the Mann–Whitney U test was used for comparisons between OLP and control groups in qRT-PCR validation and clinical sample analysis. *p*-values < 0.05 were considered statistically significant.

## 3. Results

### 3.1. Identification of Candidate Genes

**Figure 1:**
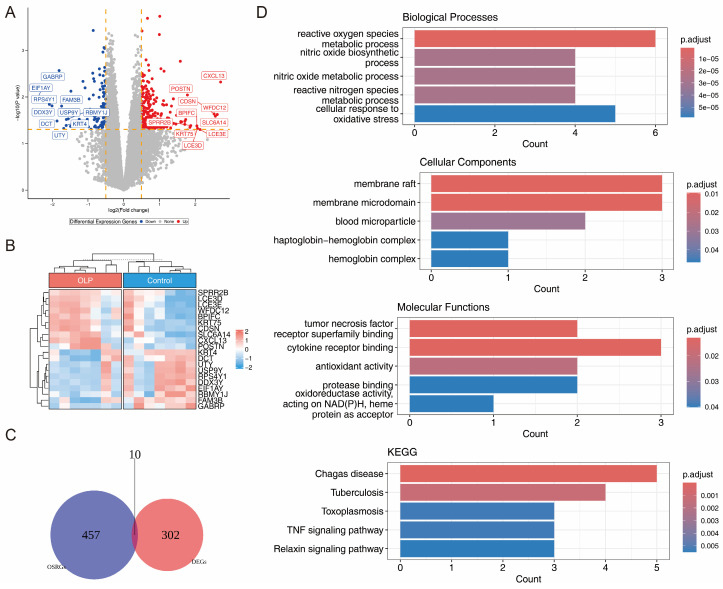
Candidate genes in OLP patients were identified from the GSE38616 dataset. (**A**) Volcano plot showing 221 upregulated and 91 downregulated genes in OLP patients. (**B**) Heatmap displaying the top 10 up and downregulated DEGs. (**C**) Venny map illustrating 10 candidate genes by overlapping the 312 DEGs with 467 OSRGs. (**D**) The top five GO and KEGG enrichment analysis terms for the 10 candidate genes. DEGs: differentially expressed genes; OSRG: oxidative stress-related genes.

Analysis of the GSE38616 dataset identified 312 DEGs, comprising 221 upregulated and 91 downregulated genes. The volcano plot and heatmap illustrated the top 10 upregulated and downregulated DEGs along with their expression patterns, respectively (Figure 1A,B). After overlapping the 312 DEGs with 467 OSRGs, 10 candidate genes were identified (Figure 1C). The 10 candidate genes were enriched in 840 GO entries and 31 KEGG pathways by enrichment analysis. These included processes related to antioxidant activity and pathways associated with tuberculosis and toxoplasmosis (Figure 1D, Tables S4 and S5). The results suggested that the candidate genes were significantly associated with oxygen metabolism and various diseases.

### 3.2. Identification of Core Biomarkers by PPI Network Construction and Analysis of Their Expression Correlations

**Figure 2:**
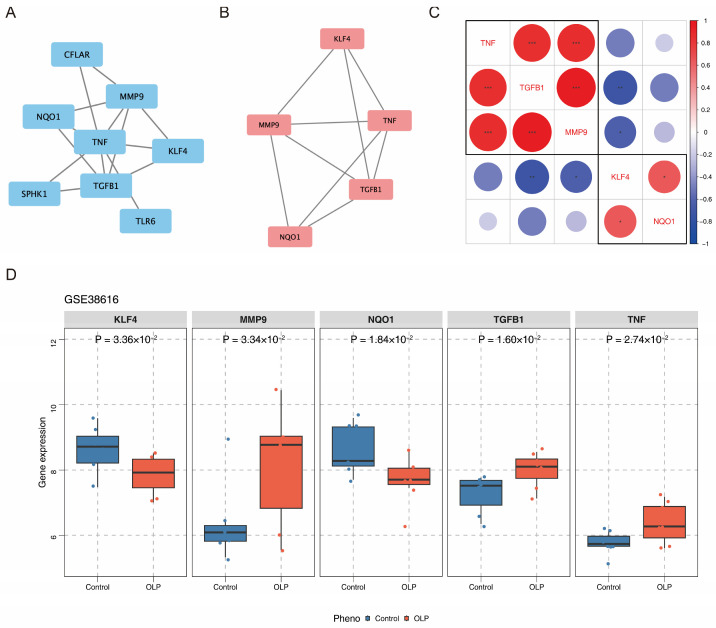
TGFB1, KLF4, TNF, NQO1, and MMP9 were deemed as biomarkers. (**A**) PPI network construction of the top 10 candidate genes, with two discrete genes were removed. (**B**) The MCODE plugin identified the key module, which contained TGFB1, KLF4, TNF, NQO1, and MMP9. (**C**) Spearman’s correlation analysis of the five biomarkers. TGFB1 and MMP9 were most significantly positively correlated, and TGFB1 and KLF4 were most significantly negatively correlated. (**D**) Boxplot showed the expression level of the five biomarkers according to the GSE38636 datasets. KLF4 and NQO1 were significantly downregulated, whereas TGFB1, TNF, and MMP9 were significantly upregulated. Statistical significance levels are indicated as follow: * *p* < 0.05; ** *p* < 0.01; *** *p* < 0.001.

After constructing the PPI network, two discrete targets were removed (Figure 2A). Genes such as TGFB1, MMP9, and TNF were highly correlated with other genes. The key module with the highest score, which was identified using the MCODE plugin, contained TGFB1, KLF4, TNF, NQO1, and MMP9 (Figure 2B). Thus, they were identified as biomarkers. In addition, Spearman’s correlation analysis revealed that TGFB1 and MMP9 were the most significantly positively correlated (cor = 0.95, *p* < 0.05) and TGFB1 and KLF4 were the most significantly negatively correlated (cor = −0.71, *p* < 0.05) (Figure 2C, Table S6). The expression analysis revealed that in the OLP samples, KLF4 and NQO1 were significantly downregulated, whereas TGFB1, TNF, and MMP9 were upregulated (*p* < 0.05) (Figure 2D).

### 3.3. Identification of Potential Pathogenic Mechanisms Through Functional Enrichment Analyses (GSEA and GSVA) of the Biomarkers

**Figure 3:**
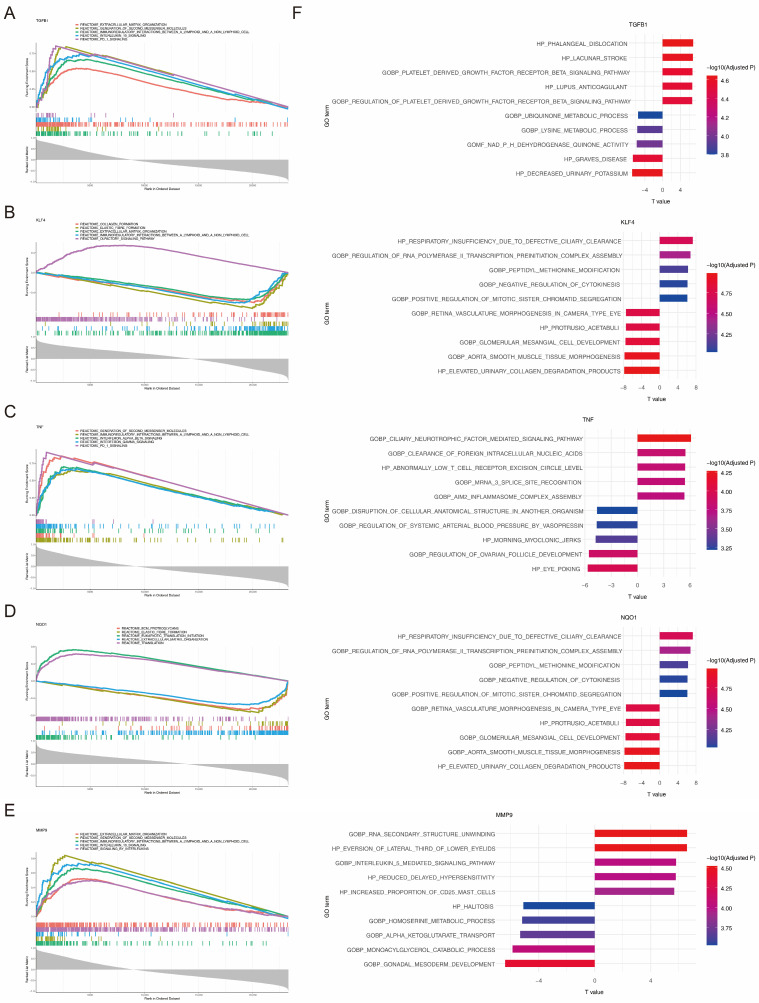
Functional enrichment analysis of the five biomarkers using GSEA and GSVA (**A**–**E**). GSEA analysis revealed the top five enriched pathways for TGFB1 (**A**), KLF4 (**B**), TNF (**C**), NQO1 (**D**), and MMP9 (**E**). (**F**) GSVA revealed the top five activated pathways and the top five inhibited pathways associated with the five biomarkers.

GSEA analysis identified 421, 370, 485, 359, and 452 significantly enriched pathways associated with TGFB1, KLF4, TNF, NQO1, and MMP9, respectively (Table S7). Among the top five enriched pathways, TGFB1, KLF4, TNF, and MMP9 all showed significant associations with immunoregulatory interactions between lymphoid and non-lymphoid cells, whereas TGFB1, KLF4, NQO1, and MMP9 showed significant associations with extracellular matrix organization (Figure 3A–E). GSVA comparing high- and low-expression groups revealed the following: for TGFB1, 1276 pathways were markedly activated, whereas 421 pathways were markedly inhibited; for KLF4, 367 pathways showed significant activation, whereas 1530 pathways exhibited significant inhibition; for TNF, 785 pathways were significantly activated and 162 pathways were significantly inhibited; for NQO1, 367 pathways were significantly activated and 1530 pathways were significantly inhibited; for MMP9, 853 pathways exhibited significant activation, whereas 342 pathways showed significant inhibition (Figure 3F, Table S8).

**Figure 4:**
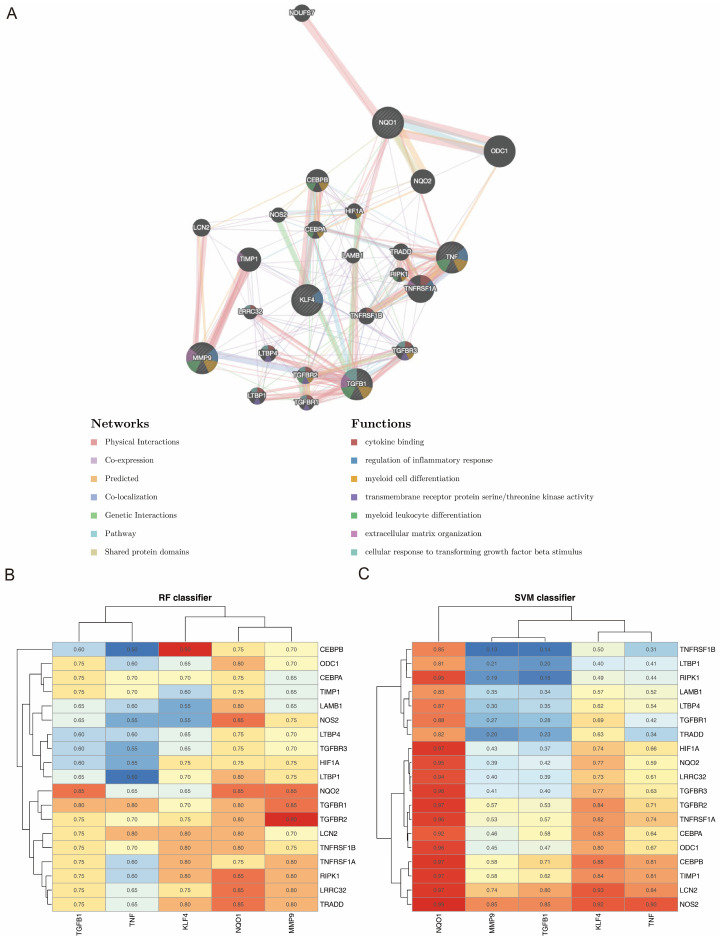
Functional enrichment analysis of the five biomarkers using the GGI network and RPIseq. (**A**) GGI network construction revealed the top 20 genes related to the functions of the five biomarkers. (**B**,**C**) The binding scores for the proteins encoded by the five biomarkers by RF classifier analysis (**B**) and SVM classifier analysis (**C**).

The constructed GGI network revealed the top 20 genes (e.g., TNFRSF1A) associated with biomarker function. These genes are involved in regulating the inflammatory response and myeloid cell differentiation (Figure 4A). In addition, the proteins encoded by KLF4 and CEBPB, as well as MMP9 and TGFBR2, exhibited the highest binding scores (RF = 0.90) in the RF classifier (Figure 4B), whereas the proteins encoded by NQO1 and NOS2 had the highest binding scores (SVM = 0.99) in the SVM classifier (Figure 4C). Thus, multiple RNA–protein interaction pairs were identified, and these interaction sites may serve as therapeutic targets.

### 3.4. Construction of a ceRNA Regulatory Network and Biomarker Expression in OSCC Through Disease-Association Analysis

**Figure 5:**
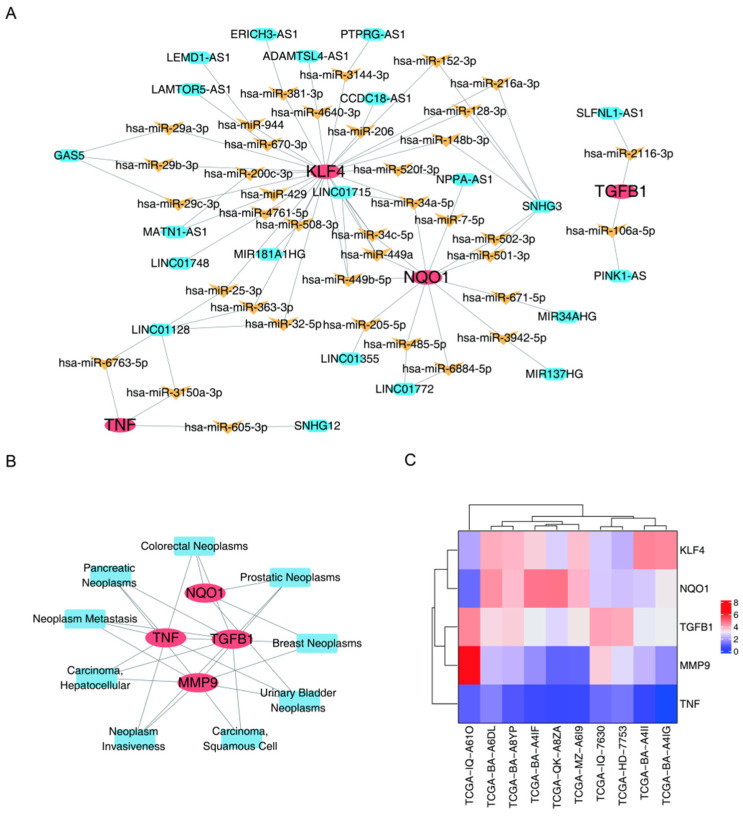
The simplified lncRNA–miRNA–mRNA regulatory network for the biomarkers. (**A**) The simplified disease–biomarker network. (**B**) Construction of a disease–biomarker network. (**C**) Heatmap showing the expression level of the five biomarkers in OSCC samples using the TCGA database. KLF4 and NQO1 showed relatively high expression, whereas MMP9 and TNF showed relatively low expression.

After intersecting miRNAs predicted by the databases, 154 key miRNAs that regulate TGFB1, KLF4, TNF, NQO1, and MMP9 were obtained (Table S9). Based on the key miRNAs, 1935 lncRNAs were predicted; however, no lncRNAs were predicted for the miRNAs targeting MMP9 (Table S10). Next, biomarkers with target lncRNAs and their miRNAs were used to construct an lncRNA–miRNA–mRNA network (Figure 5A). Of these, multiple miRNAs may target multiple biomarkers simultaneously. For example, hsa-miR449a and hsa-miR449b-5p could simultaneously target NQO1 and KLF4 and were regulated by LINC01715. Through disease-association analysis, 17, 22, 11, and 17 cancers related to TGFB1, TNF, NQO1, and MMP9 were predicted, respectively, whereas no cancers were predicted for KLF4 (Table S11). Thus, cancers related to multiple biomarkers were combined to construct a disease–biomarker network (Figure 5B), further clarifying the close connections among the biomarkers. In the OSCC samples, KLF4 and NQO1 showed relatively high expression levels, whereas MMP9 and TNF showed relatively low expression levels (Figure 5C).

### 3.5. Drug Prediction and Molecular Docking Suggest Potential Interactions with the Biomarkers

**Figure 6:**
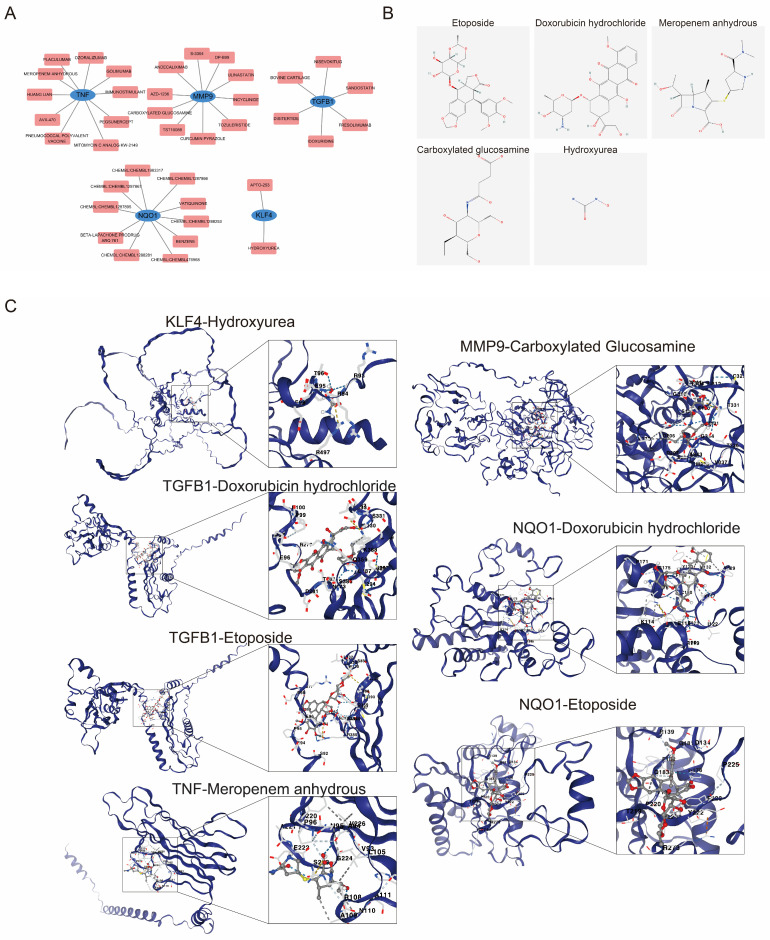
Simplified drug–biomarker interaction network predicted by DGIdb. (**A**) The drug–biomarker network revealed 38 drugs targeting the five biomarkers. Meropenem anhydrous, hydroxyurea, and carboxylated glucosamine were identified as representative candidate drugs based on their binding scores and potential relevance to the targeted biomarkers. (**B**) Structure of the key drugs. (**C**) Molecular docking of the drug–biomarker.

The drug–biomarker network predicted a total of 40 drugs targeting TGFB1, KLF4, TNF, NQO1, and MMP9 (Figure 6A, Table S12). Specific compounds were identified for the core biomarkers, including meropenem anhydrous targeting TNF, hydroxyurea targeting KLF4, and carboxylated glucosamine targeting MMP9. These candidates were selected as key drugs for further analysis based on their structural characteristics and molecular weights (Figure 6B). Molecular docking simulation indicated that, except for the binding energy between KLF4 and hydroxyurea, the theoretical binding energies of other key drugs to their corresponding biomarkers were less than −5 kcal/mol (Figure 6C, Table 2). Taken together, these results highlight the potential role of TGFB1, KLF4, TNF, NQO1, and MMP9 for treatment. Among the predicted candidates, etoposide and doxorubicin hydrochloride are categorized as cytotoxic chemotherapeutic agents. While exhibiting high molecular binding affinity, their clinical applicability for OLP management is constrained by documented systemic toxicities.

### 3.6. The Predicted m6A Modification Sites and the Shared RBP (DDX3X) of Biomarkers

**Figure 7:**
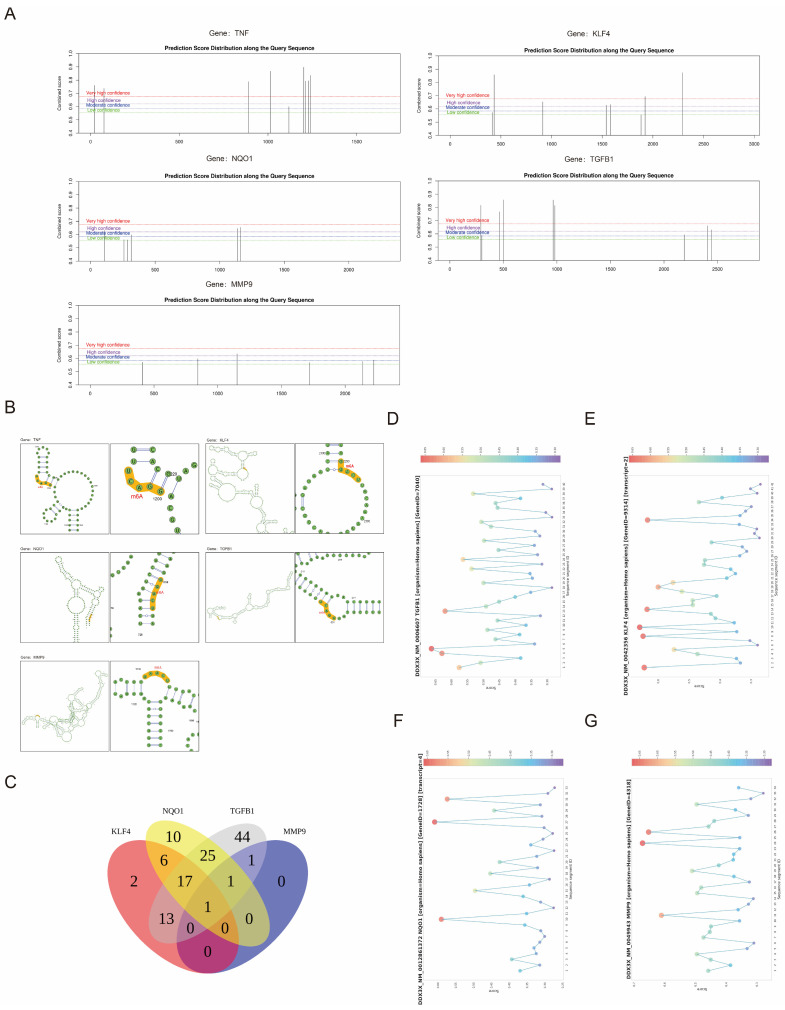
Prediction of the m6A modification sites and the shared RBP of the five biomarkers. (**A**) Prediction score distribution of the m6A modification among the query sequences. (**B**) The m6A modification sites with the highest scores for each biomarker are displayed in the RNA secondary structure. (**C**) The ENCORI database predicted the target probability of RBP for the five biomarkers. RBP may target TGFB1, KLF4, NQO1, and MMP9, but not TNF. (**D**–**G**) RBP (DDX3X) showed high-scoring RNA-binding regions with TFGB1, KLF4, NQO1, and MMP9.

In the sequences of TNF, KLF4, and TGFB1, m6A modification sites with very high confidence were identified. The m6A modification sites with the highest scores for each biomarker were displayed in the RNA secondary structure (Figure 7A,B). An analysis using the ENCORI database predicted that TNF was not targeted by any RBP. In contrast, TGFB1, KLF4, NQO1, and MMP9 were potentially targeted by the RBP (DDX3X) (Figure 7C). RBP (DDX3X) had a high binding probability with NQO1 and KLF4 for two classifiers (RF and SVM) (Table 3). In addition, RBP (DDX3X) had high-scoring RNA binding regions with TGFB1, KLF4, NQO1, and MMP9. The number of high-scoring RNA-binding regions with KLF4 was the largest (Figure 7D–G), which suggests that the interaction between RBP (DDX3X) and KLF4 at the molecular level may be closer.

### 3.7. Annotation Yielding 10 Cell Types

**Figure 8:**
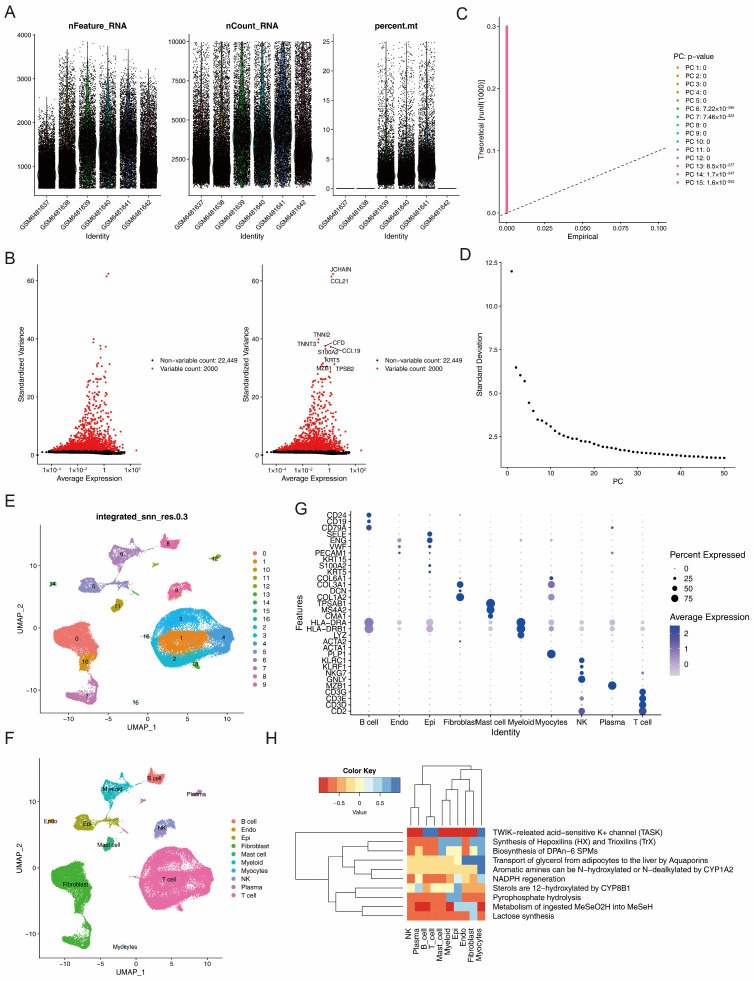
Annotation of the scRNA-seq dataset GSE211630 for OLP lesional samples. (**A**) Quality control was performed on the GSE211630 dataset. (**B**) Volcano map showing the top 2000 highly variable genes (HVGs). (**C**) Principal component analysis (PCA) was conducted on the HVGs. (**D**) Scree plot displaying the top 30 principal components (PCs) for downstream analysis. (**E**) UMAP showing 17 different cell clusters. (**F**) Ten cell types were annotated in the 17 cell clusters, namely, B cells, endothelial cells, epithelial cells, fibroblasts, mast cells, myeloid cells, myocytes, natural killer cells, plasma cells, and T cells. (**G**) The important marker genes for the different populations were identified, and high specificity was evident in the different cell clusters. (**H**) Heatmap showing the enrichment analysis of the 10 cell types. The results indicated that different cell types were enriched to varying degrees in multiple metabolic and biosynthetic pathways.

In the GSE211630 dataset, before quality control filtering, 62,357 cells and 24,449 genes were identified. Following stringent quality control, 49,097 cells and 24,449 genes were retained for subsequent analysis (Figure 8A). The top 2000 HVGs and the top 30 PCs were used for UMAP-based clustering (Figure 8B–D). All high-quality cells were segregated into 17 distinct cell clusters (Figure 8E). Through systematic annotation of the cell clusters, 10 distinct cell types were identified, which included B cells, mast cells, endothelial cells, fibroblasts, epithelial cells, myeloid cells, myocytes, natural killer cells, plasma cells, and T cells (Figure 8F). The marker genes showed high specificity across different cell clusters (Figure 8G). Furthermore, functional enrichment analysis of the 10 cell types revealed that distinct cell populations were enriched to a varying extent in multiple metabolic and biosynthetic pathways. For example, the most prominent activated pathway for B cells, myocytes, and T cells was the TWIK-related acid-sensitive K+ channel (TASK) pathway (Figure 8H, Table S13).

### 3.8. Identification of T Cells as Key Subpopulations and Analysis of Biomarker Expression Dynamics in T-Cell Subtypes and Differentiation

**Figure 9:**
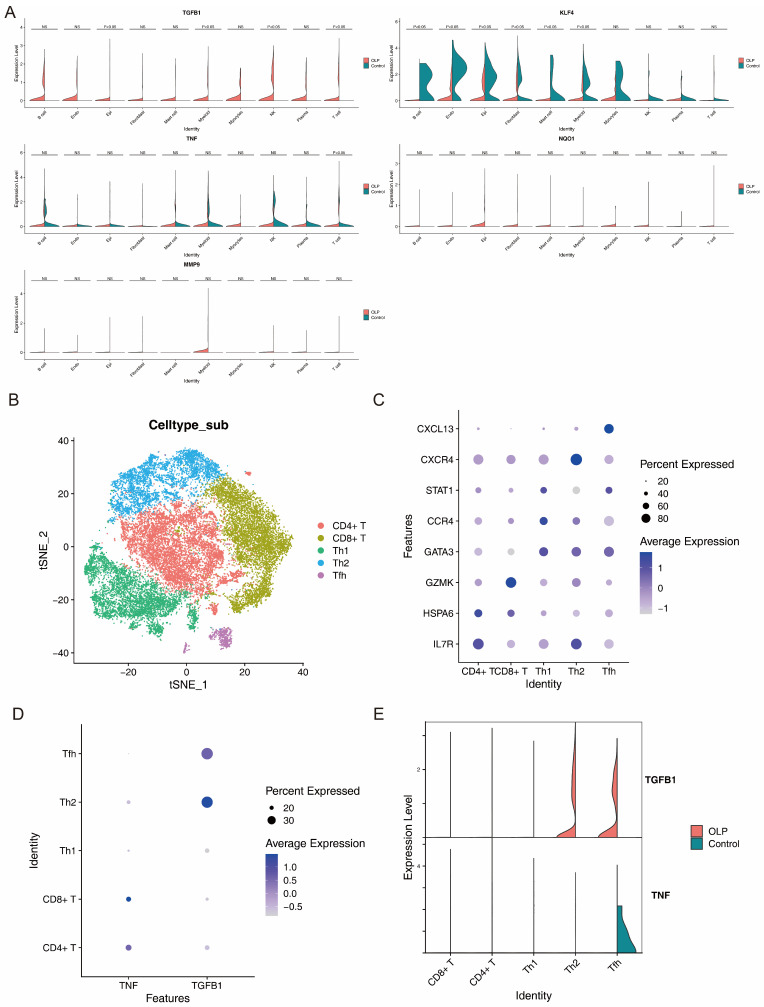
T cells were identified as key cells in the OLP samples. (**A**) The expression of TGFB1, KLF4, TNF, NQO1, and MMP9 at the cellular level was examined in the GSE211630 dataset. T cells were identified as key cells in the OLP samples. (**B**) The tSNE contained five T-cell subtypes based on the marker genes. (**C**) The marker genes are expressed in the five T-cell subtypes. High specificity of the marker genes was observed in the various subtypes. (**D**,**E**) The features (**D**) and expression levels of TNF and TGFB1 in the five T-cell subtypes. TGFB1 was highly expressed in Th2 cells and showed a significant difference between the OLP and control samples.

The expression levels of TGFB1, KLF4, TNF, NQO1, and MMP9 at the cellular level was evaluated in the GSE211630 dataset. TNF and TGFB1 were markedly upregulated in T cells from the OLP samples, whereas KLF4 was significantly downregulated in all 10 cell types without specificity. The level of MMP9 and NQO1 expression in different cell types was low, or the differences did not reach significance (Figure 9A). Therefore, T cells were considered key cells. Next, secondary dimensionality reduction clustering was performed on the key cells. Combined with the marker genes for the T-cell subtypes (Table S14), T cells were divided into five subtypes (Figure 9B). The marker genes showed high specificity for different T-cell subtypes (Figure 9C). TGFB1 was highly expressed in Th2 cells and exhibited a significant difference between OLP and the control samples (Figure 9D,E), whereas the other biomarkers were not significantly altered in the T-cell subtypes (*p* > 0.05). Single-cell analysis showed that TGFB1 was differentially expressed between OLP and control samples across specific T-cell subtypes. Nevertheless, a pseudotime trajectory was inferred based on the limited number of samples. Thus, the deduced developmental path and gene expression trends serve as a predictive model that requires further validation with larger datasets to rule out bias caused by individual variation. TGFB1 was highly expressed in Th2 cells and showed a significant difference between OLP and the control samples (Figure 9D,E); however, considering that the control group in the GSE211630 dataset consisted of a single biological sample, the differential expression patterns observed in the specific T-cell subtypes should be interpreted with caution. The limited control data may not fully reflect the baseline expression variability in a healthy population.

**Figure 10:**
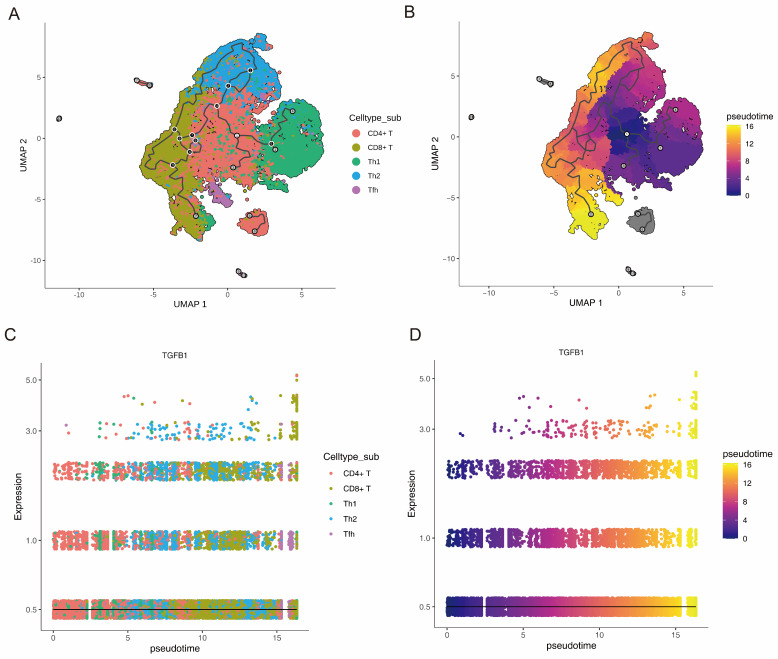
The expression of TGFB1 during T cell differentiation. (**A**) UMAP showing the five T-cell subtypes. (**B**) Pseudotime analysis of each subtype. The darker the purple color, the earlier the cell differentiation. Of these, the T cells and T-cell subtypes had 12 differentiation states, and state 1 was differentiated the earliest. (**C**,**D**) TGFB1 expression during the differentiation of T cells (**C**) and T-cell subtypes (**D**). An upward trend in TGFB1 was observed with the differentiation of the T cells and T-cell subtypes.

T cells representing different subtypes were arranged on a developmental trajectory according to the differentiation time. The darker the purple color, the earlier the cell differentiation. Of these, T cells and T-cell subtypes exhibited 12 differentiation states, and state 1 was differentiated the earliest (Figure 10A,B). TGFB1 expression showed an upward trend with the differentiation of T cells and T-cell subtypes (Figure 10C,D).

### 3.9. Validation of the Expression Levels of Core Biomarkers in OLP Clinical Samples by qRT-PCR

**Figure 11:**
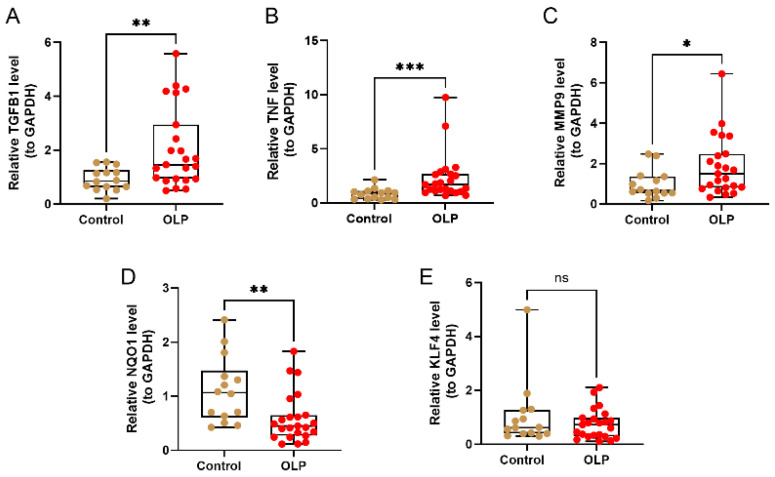
Validation of the expression level of the five biomarkers. Samples representing 23 OLP lesional lesions and 14 control tissues were subject to qRT-PCR to validate the expression of the five biomarkers. The expression of TGFB1 (**A**), TNF (**B**), and MMP9 (**C**) was significantly increased in OLP samples, and the expression of NQO1 (**D**) was significantly decreased in OLP samples. A downward trend of KLF4 was observed in the OLP samples, which was not significant (**E**). Date are presented as mean ± SEM. Statistical significance is indicated as follows: ns, not significant; * *p* < 0.05; ** *p* < 0.01; *** *p* < 0.001.

Previous studies have shown that in OLP patients, KLF4 and NQO1 expressions are markedly downregulated, whereas those of TGFB1, TNF, and MMP9 are upregulated (*p* < 0.05) (Figure 2D). Therefore, qRT-PCR was used to confirm the expression of these biomarkers in OLP patient samples. TGFB1, TNF, and MMP9 expressions were significantly upregulated, and that of NQO1 was significantly downregulated (*p* < 0.05), which is consistent with the results from the GSE38616 dataset (Figure 11A–D). Although KLF4 exhibited a downward trend in OLP samples, the difference did not reach statistical significance (*p* = 0.19) (Figure 11E), possibly because of the limited sample size or individual heterogeneity.

## 4. Discussion

OLP is a relatively common chronic inflammatory and immune-mediated disorder of unclear etiology [23]. Recent evidence suggests that OS substantially contributes to OLP development and progression, although the underlying molecular mechanisms remain to be fully elucidated [24]. In this study, five OS-related biomarkers were identified (TGFB1, KLF4, TNF, NQO1, and MMP9). Their potential functions were examined through enrichment analysis, regulatory network construction, drug prediction, and RNA modification. By integrating single-cell transcriptomic data with clinical validation, individual cell populations were determined, and biomarker expression patterns were characterized at the cellular level. Overall, the findings provide novel insights into potential OLP pathogenic mechanisms.

TGFB1, a member of the dimeric polypeptide growth factor family, serves as an important immunoregulatory mediator of T-cell function [25,26]. Single-cell analysis revealed that TGFB1 is predominantly expressed in T-cell subtypes, particularly Th2 cells, with an upward trend observed during the differentiation of T cells. These results suggest that TGFB1 regulates the imbalance of T cells in OLP by modulating the inflammatory response and cell maturation. Furthermore, the identified m6A modification sites on TGFB1 mRNA suggest a potential epigenetic layer of regulation. Such modifications might affect mRNA stability, a phenomenon observed in other contexts where m6A modification promotes the decay of TGFB1 mRNA [27]. These specific regulatory axes in OLP represent directions for future mechanistic studies to clarify how TGFB1 maintains the chronic immune microenvironment.

In addition to immune regulation, TGFB1 enhances MMP9 expressions in human mucosal keratinocytes [28]. MMP9, also known as gelatinase B, participates in extracellular matrix degradation, tissue remodeling, and physiological tissue turnover [29]. The clinical validation in this study confirmed the elevated expression of MMP9 in OLP lesions, which correlates with hallmark histological features such as basal keratinocyte degeneration and basement membrane disruption. Furthermore, increased MMP9 levels may facilitate the malignant transformation from OLP to oral carcinoma [30,31]. This indicates that MMP9-mediated tissue remodeling is a critical bridge between chronic inflammation and potential carcinogenesis in the oral mucosa.

TNF-α belongs to the TNF/TNFR cytokine superfamily and acts as a central regulator of immune system homeostasis and host defense [32,33]. This study identified increased TNF expression in OLP, particularly within T cells. TNF-α functions as an enhancer that regulates TCR-dependent CD8+ cytotoxic T-cell activation through NF-κB signaling pathways, thereby promoting T-cell proliferation and cytokine production [32,34]. This activation may directly trigger keratinocyte apoptosis, exacerbating local OLP lesions. The crosstalk between keratinocytes and T cells via TNF signaling is typical of OLP histopathology, where activated CD8+ T cells secrete cytokines that trigger apoptosis in keratinocytes through pathways such as TNF-TNFR and Fas-FasL [1,2].

In contrast to the upregulated markers, the downregulation of KLF4 and NQO1 points toward a compromised antioxidant defense system in OLP. KLF4 is involved in cell proliferation, differentiation, and maintaining epithelial homeostasis [21,35]. Its reduced expression in OLP epithelial cells suggests a failure in protective signaling, which may exacerbate inflammatory responses [36]. Similarly, NQO1 functions as a key antioxidant enzyme that scavenges reactive oxygen species and maintains neuronal redox balance [37,38]. The significant decrease in NQO1 observed in OLP lesions suggests that its functional impairment correlates with increased susceptibility to OS-mediated damage. Moreover, the high binding probability identified between the RNA-binding protein DDX3X and the transcripts of KLF4 and NQO1 hints at a post-transcriptional regulatory network that governs the oxidative response in OLP.

The oral cavity is an environment for a variety of microbes, and disturbances in this microbiota may relate to OLP development. For instance, Prevotella melaninogenica has been found to be more abundant in OLP and may contribute to the pathogenic process by activating the transcription of inflammatory cytokines like TNF-α [39]. In the context of drug prediction, meropenem anhydrous was identified as a potential candidate targeting TNF. As a broad-spectrum carbapenem antibiotic, its predicted interaction with TNF suggests a theoretical possibility of influencing the inflammatory microenvironment, although this requires rigorous pharmacological validation to confirm efficacy and safety [40].

Although TGFB1, TNF, MMP9, KLF4, and NQO1 are involved in inflammation or OS, this study provides novel insight by systematically integrating bulk RNA-seq and scRNA-seq data to define their specific roles in OLP. Unlike previous studies that focused on isolated genes, this study established a comprehensive regulatory network linking these biomarkers to specific noncoding RNAs (e.g., hsa-miR449a) and identified T cells as the precise cellular niche for their dysregulation.

In the present study, OLP lesional samples were also utilized to reconfirm the expression of these five biomarkers by qRT-PCR. Consistent with the GSE38616 dataset, the expression of TGFB1, TNF, and MMP9 was significantly increased, and that of NQO1 was significantly decreased in OLP samples. Decreased expression of KLF4 was observed in OLP based on bioinformatics; however, qRT-PCR validation did not show a statistically significant difference, although downregulation was evident. This discrepancy suggests that while KLF4 is a potential candidate derived from transcriptomic analysis, its role requires further verification with a larger cohort to rule out limitations in sample heterogeneity. Further experimental and clinical studies involving larger sample sizes are needed to examine their potential mechanistic roles in OLP.

Several limitations in the present study must be acknowledged. First, the sample sizes in both the bulk transcriptomic and single-cell datasets are relatively small, which may introduce bias during subgroup analyses. Second, while molecular docking provided theoretical insights, the in silico findings lack experimental validation (e.g., in vivo or in vitro efficacy assays). Finally, although qRT-PCR validated the expression of most biomarkers, KLF4 did not reach statistical significance in the clinical cohort, necessitating further validation in larger populations to rule out limitations in sample heterogeneity. Future studies should focus on expanding sample sizes and conducting biological experiments to confirm the therapeutic potential of the predicted regulatory mechanisms.

## 5. Conclusions

In conclusion, the aims of this study were effectively addressed through a multidimensional approach. First, systematic screening identified five core OS-related biomarkers (TGFB1, KLF4, TNF, NQO1, and MMP9), establishing a foundation for investigating the role of OS in OLP. Furthermore, mechanistic exploration revealed that these biomarkers are associated with immunoregulatory networks and OS pathways. Therefore, potential regulatory axes, such as hsa-miR449a-mediated modulation, were proposed. In addition, expanded analyses involving drug prediction and single-cell sequencing highlight the molecular binding potential of specific compounds (as theoretical models) and identify T cells as the key cellular microenvironment for biomarker expression. Ultimately, experimental validation by qRT-PCR confirmed the dysregulated status of TGFB1, TNF, MMP9, and NQO1 in clinical samples, providing empirical evidence for their role in OLP pathogenesis. In summary, while constrained by the inherent limitations of in silico predictions and sample size, this study fills a gap by constructing a comprehensive OS-related biomarker profile, thereby offering a novel theoretical reference for the precise diagnosis and targeted treatment of OLP.

## Figures and Tables

**Table 1:** Clinical features of the subjects.

	Control (*n* = 14)	OLP (*n* = 23)
Gender		
Female	8	14
Male	6	9
Age(y)		
Range	30–62	31–78
Mean ± SEM	48.93 ± 9.11	54.17 ± 11.35

**Table 2:** The binding energies of the drug–biomarker.

Biomarker	Drug	Binding Energy (kcal/mol)
KLF4	Hydroxyurea	−3.9
TNF	Meropenem anhydrous	−6.2
MMP9	Carboxylated Glucosamine	−6.4

**Table 3:** Binding probability of RBP (DDX3X) to the biomarkers.

RBP	mRNA	Prediction Using an RF Classifier	Prediction Using an SVM Classifier
DDX3X	TGFB1	0.75	0.4
DDX3X	MMP9	0.75	0.37
DDX3X	NQO1	0.8	0.97
DDX3X	KLF4	0.8	0.74

## Data Availability

Data Availability Statement: Publicly available datasets were analyzed in this study. These data can be found here: Gene Expression Omnibus (GEO) under accession numbers GSE38616 (https://www.ncbi.nlm.nih.gov/geo/query/acc.cgi?acc=GSE38616 (accessed on 13 November 2024)) and GSE211630 (https://www.ncbi.nlm.nih.gov/geo/query/acc.cgi?acc=GSE211630 (accessed on 13 November 2024)). The original clinical validation data presented in this study are available on request from the corresponding author. The access date for all URLs in Section 2 Materials and Methods is 13 November 2024.

## Supplementary Materials

The following supporting information can be downloaded at: https://www.mdpi.com/article/10.3390/biomedicines14020420/s1, Table S1: OSRGs; Table S2: Marker genes-Cell type; Table S3: The primer sequences for biomarkers and ACTIN; Table S4: GO analysis; Table S5: KEGG analysis; Table S6: biomarker-cor; Table S7: GSEA; Table S8: GSVA; Table S9: miRNAs; Table S10: lncRNAs; Table S11: cancer-related diseases; Table S12: drug–biomarkers; Table S13: enrichment analysis of the 10 cell types; Table S14: Marker genes—T-cell subtypes.

## Abbreviations

The following abbreviations are used in this manuscript:

OS	Oxidative stress
OLP	Oral lichen planus
OSRGs	Oxidative stress-related genes
MDA	Malondialdehyde
8-OHdG	8-hydroxy-deoxy
AOPP	Advanced oxidation products
PPI	Protein–protein interaction
GEO	Gene Expression Omnibus
scRNA-seq	Single-cell RNA sequencing
DEGs	Differentially expressed genes
GO	Gene ontology
KEGG	Kyoto Encyclopedia of Genes and Genomes
GSEA	Gene set enrichment analysis
MSigDB	Molecular Signatures Database
NES	normalized enrichment score
FDR	false discovery rate
GSVA	Gene Set Variation Analysis
GGI	gene–gene interaction
SVM	Support vector machine
miRNAs	microRNAs
lncRNAs	long non-coding RNAs
OSCC	oral squamous cell carcinoma
TCGA	The Cancer Genome Atlas
DGIdb	Drug–Gene Interaction Database
m6A	N6-methyladenosine
TASK	TWIK-related acid-sensitive K^+^ channel
CD8^+^Trm	Tissue-resident memory CD8^+^T
