# Supplementary material for: Identification and Mechanism Research of Oxidative Stress-Related Biomarkers in Oral Lichen Planus
Source: Biomedicines. 2026 Feb 13;14(2):420. doi: 10.3390/biomedicines14020420 (PMC12938463; doi:10.3390/biomedicines14020420)
Supplement: Supplementary file 1 [file biomedicines-14-00420-s001.zip › SupplementaryTable S3.pdf]

Table S3-Primer sequences for biomarkers and ACTIN

Primer	Sequences
TGFB1 F	CTCCTCCAGGAGACGGATCT
TGFB1 R	CTCGAGGGAAAGCTGAGGTC
TNF F	CACCACTTCGAAACCTGGGA
TNF R	AGGAAGGCCTAAGGTCCACT
MMP9 F	GTACTCGACCTGTACCAGCG
MMP9 R	ATGCCATTCACGTCGTCCTT
KLF4 F	TCTCTTCGTGCACCCACTTG
KLF4 R	CAGCCCGAGCTACAAATCCC
NQO1 F	GAGTCCCTGCCATTCTGAAA
NQO1 R	ATGTCCCCGTGGATCCCTT
ACTIN F	CCTTGAGGCTATCCAGCGTA
ACTIN R	GTTCACACGGCAGGCATACT
